# Nanoceria Attenuated High Glucose-Induced Oxidative Damage in HepG2 Cells 

**DOI:** 10.22074/cellj.2016.3992

**Published:** 2016-04-04

**Authors:** Mohammad Shokrzadeh, Hakimeh Abdi, Azin Asadollah-Pour, Fatemeh Shaki

**Affiliations:** 1 Pharmaceutical Science Research Center, Faculty of Pharmacy, Mazandaran University of Medical Sciences, Sari, Iran; 2 Department of Toxicology and Pharmacology, Faculty of Pharmacy, Mazandaran University of Medical Sciences, Sari, Iran

**Keywords:** Nanoceria, Hyperglycemia, Oxidative Stress, Cytotoxicity, HepG2

## Abstract

**Objective:**

Hyperglycemia, a common metabolic disorder in diabetes, can lead to oxidative damage. The use of antioxidants can benefit the control and prevention of diabetes side effects. This study aims to evaluate the effect of nanoceria particles, as an antioxidant, on glucose induced cytotoxicity, reactive oxygen species (ROS), lipid peroxidation (LPO) and glutathione (GSH) content in a human hepatocellular liver carcinoma cell line (HepG2) cell line.

**Materials and Methods:**

In this experimental study, we divided HepG2 cells into these groups: i. Cells treated with 5 mM D-glucose (control), ii. Cells treated with 45 mM D- mannitol+5 mM D-glucose (osmotic control), iii. Cells treated with 50 mM D-glucose (high glucose), and iv. Cells treated with 50 mM D-glucose+nanoceria. Cell viability, ROS formation, LPO and GSH were measured and analyzed statistically.

**Results:**

High glucose (50 mM) treatment caused significant cell death and increased oxidative stress markers in HepG2 cells. Interestingly, nanoceria at a concentration of 50 mM significantly decreased the high glucose-induced cytotoxicity, ROS formation and LPO. This concentration of nanoceria increased the GSH content in HepG2 cells (P<0.05).

**Conclusion:**

The antioxidant feature of nanoceria particles makes it an attractive candidate for attenuation of hyperglycemia oxidative damage in different organs.

## Introduction

LDiabetes is a metabolic disorder that affects numerous people worldwide. In this disorder, deficiency of insulin action, secretion or both causes impaired metabolism of glucose, which leads to hyperglycemia (1). Hyperglycemia is responsible for various complications such as microand macro-vascular disorders and multi-organ damage (2). The high concentration of glucose leads to oxidative stress, which plays an important role in the glucose-induced cellular dysfunction in diabetes complications. Oxidative stress is defined as an imbalance between free radical production and antioxidant defense in a biologic system. Oxidative stress is involved in aging and a number of disorders such as cardiovascular diseases, cancer and diabetic complications (3). It has been reported that irregular cellular metabolism in diabetes leads to generation of free oxygen radicals and reduction of antioxidant capacity (4,5). 

The liver is the initial organ for metabolism and regulation of glucose. Hyperglycemia has an important role in the onset of liver damage. An important strategy to protect the normal functioning of the liver and control the development of high glucose induced liver damage is inhibition of reactive oxygen species (ROS) generation. According to previous reports, oxidative stress is the biochemical trigger for hepatic dysfunction in diabetic rats (6). Apoptotic cell death is one of the cellular responses to high glucose induced oxidative stress and ROS production in mitochondria (7,8). Management of hyperglycemia by chemical drugs or insulin causes numerous problems such as fatty liver induced by insulin (9,10). An effective, economic way to manage the side effects of hyperglycemia can be the reduction of excess ROS generation by using natural antioxidants which are safe and easily accepted by cell systems (2,11). 

One of the suggested treatments for oxidative stress is the use of cerium oxide nanoparticles. Nanoceria particles show free radical scavenging activity by reversibly binding oxygen and shifting between the Ce^3+^(reduced) and Ce^4+^(oxidized) forms at the particle surface. Ceria nanoparticle ability to switch between these oxidation states is comparable to that of biological antioxidants. Nanoceria seems to have both superoxide dismutase mimetic activity (as Ce^4+^) and catalase mimetic activity (as Ce^3+^) (12). Therefore, nanoceria can act as an antioxidant and destroy free radicals (13). 

The beneficial effects of nanoceria in reducing hyperglycemia induce oxidative stress in an *in vitro* model have not been investigated. Therefore, in the present study, we evaluated the protective effects of nanoceria against high glucose-induced oxidative stress mediated cell death in HepG2 cells. 

## Materials and Methods

### Chemicals

All chemicals used were of the highest quality and purchased from Sigma Chemical Co. (USA). Nanoceria particles were purchased from Notrino Co. (Iran). Organic solvents that were of analytical grade, high performance liquid chromatography (HPLC) grade or the best pharmaceutical grade were used. 

### Cell culture and groups

This experimental study was performed on a HepG2 cell line. HepG2 cells were cultured in MEM that contained 10% fetal bovine serum (FBS) supplemented with 100 U/ml penicillin, 100 µg/ml streptomycin, and 2 mM L-glutamine in a humidified atmosphere with 5% CO_2_at 37˚C. Cells were plated for 24 hours prior to the various treatments at the indicated concentrations for the different assays. 

HepG2 cells were divided into four groups: i. Cells treated with 5 mM D-glucose (control), ii. Cells treated with 45 mM D-mannitol+5 mM D-glucose (osmotic control), iii. Cells treated with 50 mM Dglucose (high glucose) and iv. Cells treated with 50 mM D-glucose+nanoceria. 

### Cell viability assay

Cell viability was evaluated by assaying the ability of mitochondria to catalyze the reduction of 3-(4,5-Dimethylthiazol-2-yl)-2,5-Diphenyltetrazolium Bromide (MTT) to a formazan salt. 

### Measurement of reactive oxygen species generation in HepG2 cells

ROS formation was determined with Dichlorodihydro-fluorescein diacetate (DCFH-DA, final concentration 20 µM) as the indicator. The fluorescence intensity of Dichlorofluorescein (DCF) was measured using a Shimadzu RF5000U fluorescence spectrophotometer. Excitation and emission wave lengths were 480 and 520 nm, respectively. The results were expressed as fluorescent intensity per 106 cells (14). 

### Measurement of lipid peroxidation

Lipid peroxidation (LPO) was estimated using thiobarbituric acid (TBA) as the indicator (15). 

### Glutathione assay

For glutathione (GSH) content estimation in HepG2 cells, 5,5′-Dithiobis(2-nitrobenzoic acid) (DTNB) was used as the indicator. Cells were analyzed with the spectrophotometric method (16). 

### Statistical analysis

Results are presented as mean ± SD. All statistical analyses were performed using the SPSS software, version 21. Statistical significance was determined using the one-way ANOVA test, followed by the post-hoc Tukey test. Statistical significance was set at P<0.05. 

## Results

**Fig.1:**
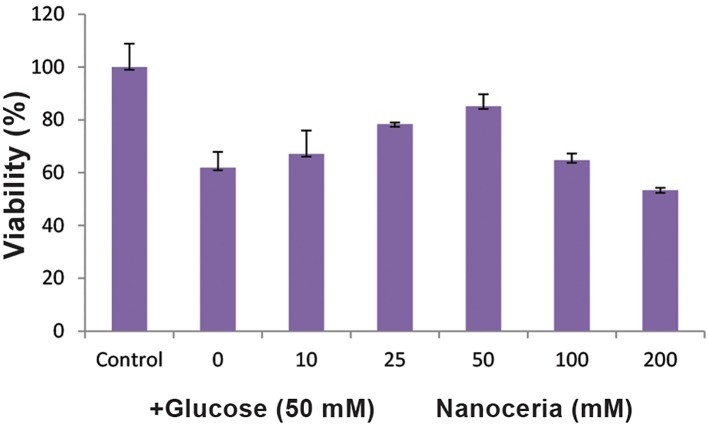
The dose-response effect of nanoceria on high glucose-induced cytotoxicity in HepG2 cells. HepG2 cells (106 cells/mL) were incubated at 37˚C with 0–200 mM nanoceria for 0.5 hours, followed by exposure to 50 mM glucose for 24, 48 and 72 hours. Cell viability was assessed using 3-(4,5-Dimethylthiazol-2-yl)-2,5-Diphenyltetrazolium Bromide (MTT) assays as described in Materials and Methods. Data represent the mean ± SD of six separate experiments.

First, we determined the best protective concentration of nanoceria against cytotoxicity induced by high glucose in HepG2 cells. As shown in Figure 1, nanoceria pretreatment (0-200 mM) significantly protected cells from the toxicity induced by high glucose (50 mM). Maximal protective effect was observed at 50 mM of nanoceria. Given this result, 50 mM nanoceria was chosen for subsequent experiments. 

**Fig.2:**
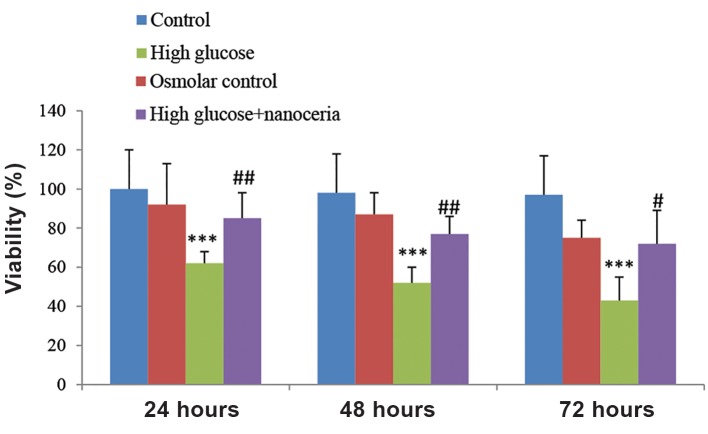
The effect of nanoceria on cell viability. HepG2 cells (106 cells/mL) were treated with 5 mM glucose (control), 45 mM mannitol+5 mM glucose (osmotic control) and 50 mM glucose (high glucose) for 24, 48 and 72 hours. The effect of nanoceria pretreatment on cell viability was assessed using 3-(4,5-Dimethylthiazol-2-yl)-2,5-Diphenyltetrazolium Bromide (MTT) assays as described in materials and methods. Data represent the mean ± SD of six separate experiments. ***; P<0.001 compared with the control group, #; P<0.05 compared with the high glucose group and ##; P<0.01 compared with the high glucose group.

As shown in Figure 2, treatment of HepG2 cells with a high concentration of glucose (50 mM) resulted in significant loss of cell viability at 24 hours (62%), 48 hours (52%) and 72 hours (43%). The osmotic control (45 mM mannitol+5 mM glucose) did not cause any cytotoxicity in HepG2 cells during the interval of 24-72 hours. Nanoceria at a concentration of 50 mM showed protective effects against hyperglycemic induced cell death in HepG2 cells at 24, 48 and 72 hours. 

**Fig.3:**
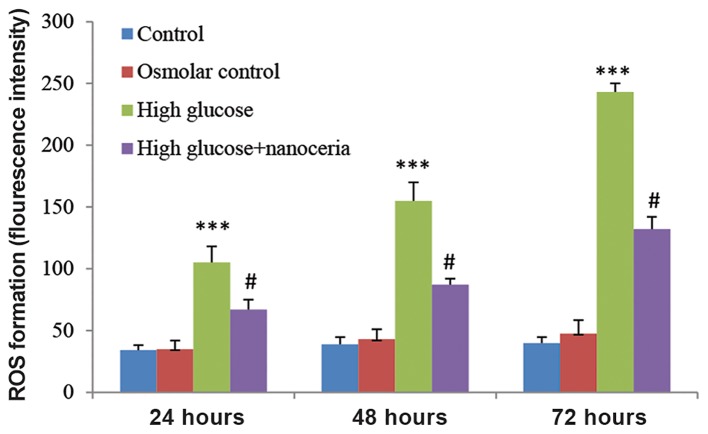
Prevention of high glucose induced reactive oxygen species (ROS) formation by nanoceria in HepG2 cells. HepG2 cells (106 cells/mL) were treated with 5 mM glucose (control), 45 mM mannitol+5 mM glucose (osmotic control) and 50 mM glucose (high glucose) for 24, 48 and 72 hours. The effect of nanoceria pretreatment on ROS formation was determined through fluorometry using Dichloro-dihydro-fluorescein diacetate (DCFH-DA) as described in materials and methods. Data represent the mean ± SD of six separate experiments. ***; P<0.001 compared with the control group and #; P<0.05 compared with the high glucose group.

The potential preventive effect of nanoceria against high glucose-induced ROS production in HepG2 cells was assayed following 24, 48 and 72 hours of incubation. Figure 3 shows that the osmotic control group did not significantly affect the intracellular production of ROS but treatment of HepG2 cells with 50 mM glucose resulted in significant increase in ROS production in a time-dependent manner. Nanoceria pretreatment effectively prevented the ROS production induced by high glucose (P<0.05). 

**Fig.4:**
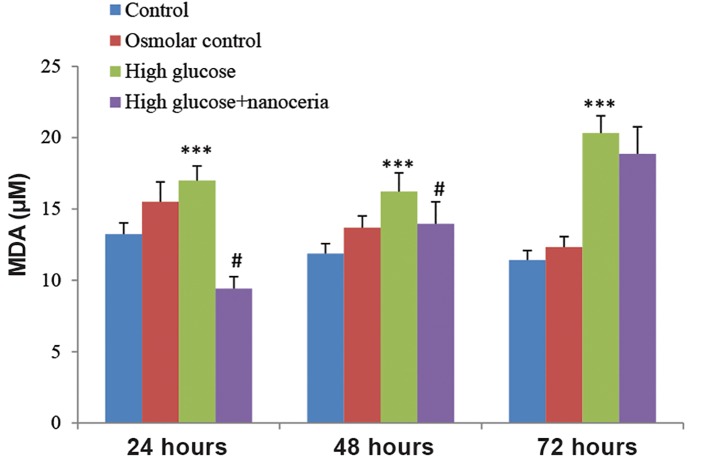
Prevention of high glucose induced lipid peroxidation (LPO) by nanoceria in HepG2 cells. HepG2 cells (106 cells/mL) were treated with 5 mM glucose (control), 45 mM mannitol+5 mM glucose (osmotic control) and 50 mM glucose (high glucose) for 24, 48 and 72 hours. The effect of nanoceria pretreatment on LPO was estimated using thiobarbituric acid (TBA) as an indicator as described in materials and methods. Data represent the mean ± SD of six separate experiments. ***; P<0.001 compared with the control group and #; P<0.05 compared with the high glucose group.

On the other hand, we observed a significant increase in malondialdehyde (MDA) concentration in HepG2 cells treated with 50 mM glucose after 24, 48 and 72 hours compared to the control group (P<0.05). HepG2 cells treated with 5 mM glucose or 45 mM mannitol+5 mM glucose showed a similar MDA concentration. Interestingly, nanoceria pretreatment significantly (P<0.05) prevented MDA elevation in HepG2 cells treated with 50 mM glucose for 24, 48 and 72 hours compared to the control group (Fig.4). 

**Fig.5:**
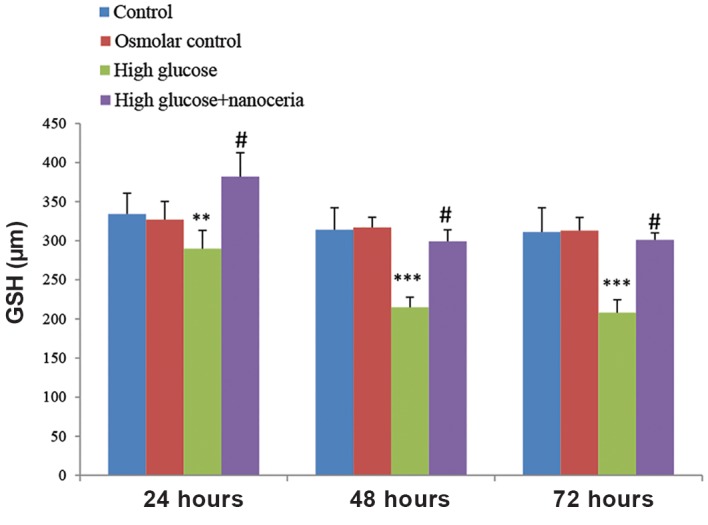
Prevention of high glucose induced glutathione (GSH) oxidation by nanoceria in HepG2 cells. HepG2 cells (106 cells/mL) were treated with 5 mM glucose (control), 45 mM mannitol+5 mM glucose (osmotic control) and 50 mM glucose (high glucose) for 24, 48 and 72 hours. The effect of nanoceria pretreatment on GSH content was estimated using 5,5′-Dithiobis(2-nitrobenzoic acid) (DTNB) as an indicator as described in materials and methods. Data represent the mean ± SD of six separate experiments. **; P<0.01 compared with the control group, ***; P<0.001 compared with the control group and #; P<0.05 compared with the high glucose group.

We observed a significantly decreased GSH content (main intracellular antioxidant) in HepG2 cells treated with 50 mM glucose for 24, 48 and 72 hours compared to the control group (Fig.5). HepG2 cells treated with 45 mM mannitol+5 mM glucose did not show any significant change in GSH content compared to the control group. Pretreatment of HepG2 cells with nanoceria particles significantly reversed the GSH oxidation induced by 50 mM glucose (P<0.05). 

## Discussion

In this study, we used a HepG2 cell line and high concentration of glucose as an *in vitro* model of glucose toxicity in liver tissue. A low concentration of glucose (5 mM) has no significant effect on cell viability compared with the untreated group (data not shown). However, we observed a significant reduction in cell viability when the HepG2 cells were incubated with a high concentration of glucose (50 mM). Numerous studies have used a high concentration of glucose (50 mM) as an *in vitro* model for investigation of hyperglycemia induced toxicity which simulated the *in vivo* condition of diabetic ketoacidosis observed in acute or untreated diabetes (17,20). 

Hyperglycemia induced-ROS production in various cell types has a key role in the pathogenesis of diabetic complications (21,23). LPO and protein oxidation can lead to membrane damage, impairment of ATP production and other vital functions in the cell. Finally, accumulation of these damages results in initiation of death signaling in the cell through necrosis or apoptosis that leads to tissue damage (24,25). 

Initially, we evaluated an oxidative stress marker after exposure of HepG2 cells to hyperglycemic conditions. As shown in the results, we observed elevated ROS production, along with LPO and GSH oxidation after exposure to 50 mM glucose compared to 5 mM glucose, or 45 mM mannitol+5 mM glucose treated HepG2 cells. Interestingly, the increase in oxidative stress paralleled the reduction of cellular viability that clearly showed the role of oxidative damage in hyperglycemia-induced cell injury. These data confirmed previous reports about the role of glucose induced oxidative stress in diabetes complications (20,26,27). It has been reported that there is a direct relation between the rate of development of complications in type 2 diabetic patients with elevation of LPO and plasma GSH and GSH-metabolizing enzymes (28,29). 

It is obvious that the most effective means for decreasing diabetes complications is tight glycemic control, however in the majority of cases this control cannot be reached (11,30). Instead of additional therapy the use of antioxidants is critical for suppressing the pathologic pathways that lead to hyperglycemia induced complications (22,31). 

Currently, nanoceria has gained attention as a potentially new agent for treatment of disorders that involve oxidative stress such as stroke, ischemia and diabetic complications (32,35). It has been shown that nanoceria could mimic both superoxide dismutase activity (in the form of Ce^4+^) and catalase activity (in the form of Ce^3+^) (36,37). We presumed that antioxidant therapy with nanoceria might be a good therapeutic approach for controlling hyperglycemic-induced oxidative damage in a HepG2 cell line. 

As indicated in the results, nanoceria at a concentration of 50 mM significantly promoted cell viability in the HepG2 cell line after exposure to a high concentration of glucose (50 mM). This effect was probably from inhibition of glucoseinduced ROS formation. We measured the rate of ROS formation in HepG2 cells that showed a significant increase in the hyperglycemic group compared to the control group. Interestingly, nanoceria prevented glucose induced-ROS formation because of antioxidant properties. Nanoceria significantly inhibited LPO in HepG2 cells which was promoted by hyperglycemia. 

We assayed cellular GSH as the main cell antioxidant that could scavenge hydrogen peroxide and other ROS (38). Our data showed that nanoceria significantly reversed GSH oxidation induced by 50 mM glucose. 

The protective role of antioxidant against diabetes induced oxidative damage was shown in various studies. Administration of vitamin C in older type 2 diabetic patients inhibited plasma free radical formation and increased cellular GSH content (39). Also, numerous studies examined the beneficial effects of vitamin E in diabetes models (40). Vitamin E administration corrected diabetes-induced changes in antioxidant enzymes in different organs. These effects were more pronounced when the combination of vitamin E with another antioxidant, stobadine, were used (41). 

In another study, treatment with red wine significantly prevented the streptozocin-induced oxidative stress in the brains of rats (42). Administration of green tea extract showed benefits in decreasing serum and hepatic MDA concentrations and increased the total antioxidant capacity in diabetic rats (26). In another study, exposure of primary rat hepatocytes to a high concentration (40 mM) of glucose led to decreased cell viability, enhanced ROS formation and depletion of the antioxidant content of hepatocytes. However treatment with Morin, a dietary flavonoid, has been shown to reverse high glucose induced oxidative stress and cell death in primary rat hepatocytes (2). Under hyperglycemic conditions, the liver is considered very sensitive to oxidative damage, which in a chronic state could lead to liver cell damage in diabetic patients (35). Thus, antioxidant therapy could be used as a therapeutic strategy for attenuating hyperglycemia side effects. 

## Conclusion

We have shown the protective effects of nanoceria treatment against hyperglycemia-induced toxicity in HepG2 cells which was probably mediated by its antioxidant properties. 
